# Childbirth experiences and their derived meaning: a qualitative study among postnatal mothers in Mbale regional referral hospital, Uganda

**DOI:** 10.1186/s12978-018-0628-y

**Published:** 2018-11-03

**Authors:** Josephine Namujju, Richard Muhindo, Lilian T. Mselle, Peter Waiswa, Joyce Nankumbi, Patience Muwanguzi

**Affiliations:** 1 Department of Nursing, Makerere University, College of Health Sciences, P.O Box 7072, Kampala, Uganda; 2 Department of Clinical Nursing, Muhimbili University of Health and Allied Sciences, Nursing and Midwifery Services Muhimbili Academic Medical Centre, P.O. Box 65427, Dar es Salaam, Tanzania; 3 Department of Health, Policy, Planning and Management, Makerere University, College of Health Sciences, School of Public Health, Kampala, Uganda; 4 Global Health Division Karolinska Institutet, Sweden and Leader Makerere University Maternal and Newborn Centre of Excellence and the INDEPTH Network Maternal and Newborn Health Research, Stockholm, Sweden

**Keywords:** Childbirth, Experiences, Meaning, Postnatal mothers, Uganda

## Abstract

**Background:**

Evidence shows that negative childbirth experiences may lead to undesirable effects including failure to breastfeed, reduced love for the baby, emotional upsets, post-traumatic disorders and depression among mothers. Understanding childbirth experiences and their meaning could be important in planning individualized care for mothers. The purpose of this study was to explore childbirth experiences and their meaning among postnatal mothers.

**Methods:**

A phenomenological qualitative study was conducted at Mbale Regional Referral Hospital among 25 postnatal mothers within two months after birth using semi-structured interviews and focus group discussions and data was thematically analyzed.

**Results:**

The severity, duration and patterns of labour pains were a major concern by almost all women. Women had divergent feelings of yes and no need of biomedical pain relief administration during childbirth. Mothers were socially orientated to regard labour pains as a normal phenomenon regardless of their nature. The health providers’ attitudes, care and support gave positive and negative birth experiences. The Physical and psychosocial support provided comfort, consolation and encouragement to the mothers while inappropriate care, poor communication and compromised privacy contributed to the mothers’ negative childbirth experiences. The type of birth affected the interpretations of the birth experiences. Women who gave birth vaginally, thought they were strong and brave, determined and self-confident; and were respected by members of their communities. On the contrary, the women who gave birth by operation were culturally considered bewitched, weak and failures.

**Conclusion:**

Childbirth experiences were unique; elicited unique feelings, responses and challenges to individual mothers. The findings may be useful in designing interventions that focus on individualized care to meet individual needs and expectations of mothers during childbirth.

## Plain ENGLISH summary

Childbirth experiences are the women’s personal feelings and interpretations of birth processes. Birth experiences to some women have meant hard work, exciting lovely event and to others it is a stressful, exhausting and unpredictable experience. Negative experiences have been associated with poor support and care, fear, excessive pain, discomfort and undesirable outcomes. Participating in making decisions regarding childbirth care and being supported by healthcare providers gives a positive memory and increases the woman’s confidence and love for the baby and better adjustment to motherhood. Understanding the women’s childbirth experiences and their meaning is important in providing socially acceptable individualized care during and after birth. In Uganda, few studies on childbirth experiences of mothers and their meanings have been done. This study explored childbirth experiences and their meanings among women within two months after giving birth. Twelve women were interviewed one on one and thirteen women in two groups of seven and six in Mbale Regional Referral Hospital, in the Eastern part of Uganda. The women reported unique experiences of labour pains, they had social orientation on labour and gave different views on pain management during birth. Negative and positive attitudes and care by service providers were described and the social support from the significant others was noted as a source of comfort and encouragement. The personal women’s and society’s interpretations of birth experiences focused on the type of birth undergone. The vaginal birth meant braveness to some mothers and caesarean birth was associated with witchcraft and weakness of a woman. In conclusion, the individual mothers had unique childbirth experiences that required service provider’s understanding and personalized care.

## Background

Childbirth is a significant event in a woman’s life and a transition to motherhood. Childbirth experiences are the subjective psychological and physiological processes, influenced by the social and environmental factors [1]. Birth experiences elicit uncertainties of the next destination with feelings of inabilities [2]. Labour pain has been regarded as a “well kept – secret” whose true reality cannot be explained until you go through it causing fear and emotional upsets [3]. Childbirth is perceived as a paradox of moments of sadness and disappointment initially and joy crowns its end if a baby is alive [3]. The interpretations of birth experiences further include hard work, exciting intimate event and a stressful, exhausting and unpredictable phenomenon [4]. To some women, giving birth is life itself, a fulfillment of God’s plan and the law of procreation and a turning point between death and life for the woman and her baby [5].

Childbirth experiences could be both positive and negative. Negative experiences are characterized by fear, excessive pain, poor support and care, discomfort and undesirable outcomes [6–8]. The negative experiences of medical interventions like epidural analgesia, induction of labor and instrumental vaginal delivery have been found to be associated with post-traumatic stress, fear of childbirth, reduced child care and emotional upsets among women [7, 9]. The positive memories of being in control over the situational happenings and the decisions on care coupled with the healthcare providers’ support are said to enhance self-confidence with feelings of accomplishment and better adjustment to motherhood [9–11]. The positive birth experiences are thus said to improve the bonding between the mother and the baby [12–14].

Understanding the women’s childbirth experiences and their meaning is crucial in the provision of individualized and culturally sensitive care during and after childbirth [15–17]. A number of studies have described women’s childbirth experiences and their meaning but these studies were done in the developed world. In Uganda, studies on childbirth experiences of mothers and the perceived meanings are scarce. This study therefore, describes the childbirth experiences and the perceived meanings among postnatal mothers seeking postnatal services at Mbale Regional Referral Hospital in Eastern Uganda to broaden the information base for appropriate intervention development and individualized care during childbirth.

## Methods

### Study design and setting

A phenomenological qualitative research design was used. Phenomenological qualitative research is an approach that describes life experiences and gives them meaning [18]. The design allows exploration of participants’ experiences, perspectives and feelings, in depth, through a holistic framework. Childbirth is a lived experience to women whose truth and reality is deeply embedded in the lives of those that have experienced it [19, 20]. In this study, it was specifically used to explore experiences, feelings and perspectives of women who had given birth. The study was conducted at Mbale Regional Referral Hospital (MRRH) located in Mbale Municipality, Northeast of Kampala, Uganda. MRRH is a public hospital with 500 bed capacity. The hospital serves 13 districts (with about 4 million people in the region) and about 800 women give birth per month in this hospital. Specifically, the study was carried out at the Young Child Clinic (YCC), one of the clinics run by the Department of Obstetrics and Gynaecology in MRRH. The clinic had four certified health care providers including 3 midwives and 1 public health nurse. It offers immunization, health education, monitoring growth and development to children under 5 years; postnatal care services, HIV counseling and testing, and referral services for example HIV positive mothers and babies were being referred to the HIV clinic for care. On average, 35 mothers and babies were attended to at this clinic per day.

### Participants and recruitment

Purposive sampling was used to recruit participants. Purposive sampling allows a researcher to get rich information to a particular research question [21]. The researcher oriented the staff of the YCC on the study and worked with the midwife in charge of the clinic to identify potential participants. The inclusion criteria were the postnatal mothers who gave birth within two months with live babies and those that were able to communicate in English, Luganda or Lumasaba. Luganda however, which all participants understood and spoke fluently was preferred in the two discussions. After receiving the services they had come for, all the potential participants were requested to meet with the researcher. She explained to them the study, its aim and how it was going to be conducted including their rights and the principles of confidentiality. Those who agreed to take part in the study gave written consent and a suitable place (with privacy) at hospital was arranged for an interview or discussion.

### Data collection

To build the credibility and better understanding of the childbirth experiences two methods of data collection were used; the semi-structured interviews (SSI) and the focus group discussions (FGDs).

### Semi-structured interviews

Twelve (12) semi-structured interviews with postnatal mothers were conducted in Luganda and English by the first author at YCC in a quiet room adjacent to the clinic. The saturation was reached with 12 interviews where the answers from mothers seemed to repeat information gained earlier with little new information [22]**.** A semi-structured interview guide with open ended questions and probes were used to explore and understand better the issues as they emerged [23] and elicited broader and deeper views from participants. All the interviews were audio recorded and lasted within 30 min.

### Focus group discussions

Two FGDs with postnatal mothers were conducted. The discussion groups included 6 and 7 postnatal mothers. The first author moderated the discussions and notes and non-verbal clues were taken by the assistant, the clinic midwife who was not involved during the participants’ recruitment process. The FGD guide used centered on the mothers’ childbirth experiences and their meanings. The discussions were held in Luganda, the local language of instruction in schools, spoken and preferred by all participants and all the discussions were audio recorded with permission from participants. The discussions lasted between 60 and 80 min.

### Data analysis

Thematic analysis guided the analysis of data. All the audio recorded interviews and discussions were transcribed verbatim and were translated from Luganda to English. The English translated transcripts were reviewed and edited to ensure correct interpretation of the mothers’ accounts. The analysis procedure included familiarization with the material through careful reading of sentence by sentence for many times, identification of the codes, searching for subcategories, formulation, revision and interpretation of themes. Phrases and sentences related to the mothers’ experiences of childbirth were coded in the margin of the transcript sheets. The coding was predominantly close to the text using mothers’ own descriptions. The codes with similar content were then brought together into sub-categories and themes. The authors discussed and reflected on the interpretations of the mothers’ descriptions of their childbirth experiences and agreed on the themes. Anonymous quotes were used to illustrate the facts.

### Methodological considerations

Qualitative researchers suggest the use of credibility, transferability, dependability and conformability as methods to ensure trustworthiness in qualitative studies [24, 25]. The researcher ensured credibility through use of multiple data collection methods (focus group discussions and in depth-interviews) which allowed triangulation of findings. Also the researcher established good rapport through having prolonged engagement in the field, used Luganda language (during FGDs) to build trust with participants. Bracketing was done through the researcher’s honest self-examination of the values, beliefs, interests and prior experiences. These were noted down and kept at the back of the mind right from proposal development through data collection, analysis and report writing to ensure that the findings were the views of the participants and not the researcher’s imaginations.Dependability and conformability were promoted through inquiry audit where by the researchers reviewed and examined the research process and the data analysis in order to ensure that the findings were consistent. Further, the thick description of the phenomenon under study, the purposive sampling used, the data collection methods that were employed and using participant’s own words during analysis and write up enhance the understanding of childbirth experiences and will allow for others to determine its transferability to other contexts [26]**.**

## Results

The 25 women who were interviewed about their childbirth experiences described themselves as housewives (*n* = 7), teachers (*n* = 6), business women (n = 6), students (*n* = 2), a journalist, an administrator, a peasant and health information assistant (*n* = 1). They were between 18 and 33 years of age, 88% (*n* = 22) were married; 88% (n = 22)gave birth in the health facilities and 12% delivered at their homes or from the traditional birth attendant. Their parity ranged from one to five and the majority were Christians (Born again (32%), Protestants (24%), Catholics (12%), the rest were Moslems (32%).

From the 12 semi-structured interviews and 2 focus group discussions, five (5) themes emerged. These included the Childbirth experiences (Labour pains and management, Institutional care and support, Childbirth fears and Social support) and the Meaning attached to childbirth experiences (Individual and Cultural interpretations). The Women reported diverse trends in their birth experiences that provided the differences and the basis for emphasis. Numbers were used to identify participants during group discussions instead of names for privacy purposes and fictive names are used in the presentation of quotes.

### Childbirth experiences

#### Labour pains and management

The memory of labour pains remained in the minds of almost all women and formed the basis for their stories. The women’s birth pain experiences were viewed and expressed in terms of intensity, duration and patterns. The severe labour pains to some women were characterized by the temporary moments of confusion and loss of understanding as one mother expressed:

*This is the 4*^*th*^ *born, other children never pained me like this one,…contractions were very strong and I reached a point when I did not understand well, they just lifted me up on to the bed. ….I just found myself delivered. After delivering, my senses came back* (Zaifa, 27 years, a mother of 4)

Rose who experienced abnormal labour patterns also said:

*Labour for my last born was hard, because the pains were much and later the contractions stopped. …..later the midwives put on a drip for contractions (Rose, 30 years, a mother of 4).*

Some womenexperienced labour for a long duration and its effects were reported with dismay, frustration and loss of hope as one mother explained:

*…the contractions pained me for 3 days… I went back, they examined me … I was still not ready. I walked and walked …. I went back at 6.00pm (second day, I was still not ready. I went back at 10.00 p.m, they told me I was not ready. I went back at midnight, the midwife told me, aaaah we are fed up with you, you go and walk so that the baby can move down, …the body was feeling like a metal, I said this time I am dead (*Eseza, 23years, a mother of one)*.*

The labour pain aspect of childbirth is one dimension that is given attention in various ways. In this study, the women were socially oriented to view labour pains as a normal phenomenon regardless of intensity, duration and varying patterns. This psychological care in preparation for birth provided consolation, hope and encouragement as narratives below indicate:

“*My attendants told me, contractions pain but you must be strong, and when time comes for the baby to come out, the baby itself will force you*” (Shifa, a 28 year old mother of 2).

*“Getting much pain happens and is human and normal to a woman, but it is God who gives you the life and energy”* (Rehema, a 33 year old mother of five)*.*

Labour pain management elicited mixed feelings from participants. Some women believed childbirth is natural and therefore should be left to take the natural course, while others felt it was necessary to reduce on the birth pains through medication if it was possible. According to the women’s descriptions, none of them received biomedical painkillers during birth and one mother explained:

*Next time I deliver, I would not want to have too much labour pains like the ones I went through. If that medicine was there, I would feel like having such a drug to reduce the pain* (Christine, 25 years, a first time mother).

Some women doubted as to whether medicine could have an effect on a natural process like labour pains as they had no prior knowledge to the intervention

*“I think no need of medicine, because it is natural. I think even if they give you some medicine for pain, contractions would still come because the baby has to come out. I think the drugs cannot reduce those pains…every other woman goes through that”* (Irene, 26 years, a primepara)*.*

#### Institutional care and support

The experiences of women regarding care and support received in the health facilities varied from positive by some mothers to negative and non-satisfying by others. Regarding institutional **births,** majority of women had **given birth** from the Regional Referral Hospital apart from one woman who had delivered from a private maternity centre. The women’s comments generally centred on the attitude of service providers, the interpersonal communication, the physical and psychological support and how the labour complications were managed. For those who felt good about their labour experiences described them in form of the good reception and attention given to them, the physical and psychological support through counseling; being listened to and having been given appropriate management to the complications as some mothers narrated:

*The midwives were good…. even when they were attending to other people, when I would also call her (musawo) meaning a health care provider, also come and help me, She would not shout at me, would say let me come, I am hearing. I used to hear that they shout at people but for me they never shouted at me. (*Christine, a 25 year old mother of one).

Another one said:

*The midwives welcomed me well, examined me … delivery time had not reached. I told them, basawo, I am sick, (HIV positive) they said you have done a good thing to tell us…, they said don’t fear. Some hide and do not tell us. ….when the time reaches you will push well and in case something wrong happens, we will help you. When time came, the midwife helped me to climb the bed. After delivery, I bled a lot … quickly they ran and gave me an injection and blood stopped. … weighed the baby, wrote treatment and gave the syrup for the baby (*Rehema, a 33year mother of 5*).*

As some mothers expressed their birth experiences with confidence and trust in service providers, to others it was a moment of reflection on the sadness, suffering and agony they went through. The women described experiences of non- caring attitude, limited technical care and support; quarreling and being rough to them. Rose,one of the mothers that was cared for by the morning and evening ward staff described her experience with the midwife of the 2nd shift:

*…. I called her that I feel like something pushing me, she never bothered. She said, “Keep quiet, for you, you are making yourself tired for nothing, you are not going to deliver now”. I forced to deliver myself, what can I do? (Voice tone lowered) I said that if I relax I would lose my baby…. When the midwife reached, the baby was out and a lot of water (liquor) had poured on the baby. She (midwife) got annoyed, quarreled…. She cut the baby’s cord from that water yet I normally see midwives cut the cord from the mother’s abdomen. I felt too bad.*

The practices reflected by some health care providers were unethical and violated the rights of the clients as one mother who was slapped during the time she pushed her baby narrated:

*… the midwife told me to push. I tried to push and push, she was even slapping me telling me to push. The old woman (attendant) had given the midwife some money, now she was on my “bamper” (on my neck). “I have told you to push the baby with slaps”*

(Ruth, a 20 year old mother of 2)

Giving birth by caesarean was a hurdle. One mother who was taken to the theatre for the caesarean birth experienced delay to be operated as theatre was unready causing her stress and anxiety. This was heighted by being left exposed naked, a factor that compromised her privacy as she lamented:

*….now you are under stress and you feel like… they have to do it (operation) immediately. “They take you in the theatre room, you again spend some good time there when they are still organizing. It was too bad, ….now even you feel stress is coming back again , you try to console yourself , you control…As you wait, you are naked, you are exposed there… (laughed while shaking her head)… you know how funny it is. ..you are nude. It was not good, privacy was not enough”.* (Caroline, a 30 year old mother of 3 children).

Many women were giving birth for the first time and as such needed more information regarding labour and its proceedings. Women noted limited effective communication and sharing of information by the service providers. The non-involvement of a mother in decision-making regarding her care resulted in a number of unanswered questions as one woman explained:

*….. the doctor told me, Irene, with you, you are just going for an operation. …I had to break down… because I was like what has gone wrong? ... why not me to deliver like other women? ….they are telling me I am going for an operation but they are not telling me the cause! Doctor Just told me, the baby was big. He then used some medical language that I did not understand. She did not convince me as to why I should have an operation… they were talking alone!* (Irene, a first time mother).

### Social support

The mothers according to their narratives appreciated the presence, proximity, the physical and psychological support from their birth companions that were basically family members (the mums, sisters, mothers-in law, aunties, husbands) and friends. Physically, the women were supported by giving them food and drinks like tea, they were supported to walk around before reaching second stage of labour and their backs were rubbed to provide relief during contractions by their birth companions.

Eseza a 23 year old mother of 3 recounted:

*Attendants (birth companions* ***)*** *helped me to get tea to drink, they kept around, and during that time when I could get the contractions, they could support me at the back, rub it, I could get some relief of about one minute.*

Irene, a 26 year old primepara, who was supported through counselling by a relative before a caesarean birth recalled:

*“At that time when I broke down,… my sister in law and mother in law were there for me. They tried to counsel and consoled me but still it wasn’t an easy moment”*

The male partners were involved in intrapartum care of their spouses at different times and in various ways. Although their (male partners’) physical presence was registered at the hospital, their participation in real care was minimal. One mother whose husband was in hospital but at a distance from the maternity unit commented:

*My husband was not near, had feared and moved away. I never wanted him to be near because when you are in labour the face changes, you may say that it is this one who brought the problems and get annoyed with him (laughed)* (Shifa, a mother of 2).

Another mother whose husband fully participated in the processes of the birth of their baby by being present at the side of his partner and providing physical and psychological support during the pushing time gave her story:

*When I came back to the labour ward they told me “the baby has reached you push”, I was not feeling any energy. ….my husband helped me, held me and he never feared. When the baby was coming out, he told me that “bambi” (meaning my friend) push more,the head is coming, add in more effort. … I felt good, I liked it so much because he gave me support,and he was there (Faith, a mother of one).*

#### Childbirth fears

Childbirth is a moment of unpredictable “next” in terms of the outcome of the baby and mother both to those who give birth normally and by caesarean birth. In this study, these experiences were worsened by giving birth by a caesarean resulting into mothers’ fear, anxiety and loss of hope for survival as one woman recalled:

*It is hard when you are going for a cesarean birth, you are always worried. …… you are not sure of what is going to happen there….. in such situation the mother is not sure of the baby’s survival and her own wellbeing; or she might come out with complications. Yah…it is like you are going for trouble when you are seeing (mother laughed) (*Catherine, a 30-year mother of 3, with 3 previous scars)*.*

The situation can be more terrifying to those who are giving birth for the first time. Irene, a first time mother had refused a caesarean birth for fear of losing her life in theatre until all hope of delivering normally was lost.

*The doctor told me, “Irene, with you, you are just going for an operation at 10am”. ….I had to break down, I did not know … “if others can push normally, why not me?” At 10 a.m I refused the operation. I told them I don’t want, give me time ……I had feared the theatre because with me I knew whoever goes to theatre, does not come back. They just die like that. I imagined very many things*

Multidimensional sources of fear were reported in this study. One mother who experienced excessive bleeding and a retained placenta regretted the decision she had made of delivering at home: “being with aunt alone I feared, I felt my life was going and regretted not having been in hospital”. The mistreatment of women by health care providers was sofrightening that one mother vowed never again to deliver in that hospital as she expressed it:

*“... I forced to deliver myself”….in that difficulty situation I went through, I got scared, I feared the midwives of the main hospital (government). I feared too much, I said next time when I deliver, at least I go to private but not go back to main hospital.* (Rose, a mother of 4 children)*.*

The negative stories (the past experiences by other mothers) about the institutional care were noted to be far reaching and a source of childbirth fears regarding the place of delivery. One mother who presumed and perceived hospital care negatively resorted to practices that undermine the quality of birth outcomes as she gave birth at the TBA’s home and gave her account:

*“I refused going to hospital. I had no appetite of going there. What I hear threatened me. I hear in the hospital they don’t care about you, you care for yourself. ……..You are seeing this one is complaining, that one grumbling, another one is dying, I avoided such things. I said at least, for me let me die from here (TBA’s) and those other ones die there in hospital”* (Amina, an 18-year-old primepara).

### Meaning attached to childbirth experiences: The individual woman’s and societal interpretations

#### The individuals’ childbirth experience meaning

The individual women interviewed had varying personal interpretations of their birth experiences. The sense made out of it was determined by what preoccupied the woman at that time of labour, the transpirations of the day and the outcomes. The supremacy of God in the event of childbirth was strongly expressed by many women but in union with self-belief and determination of an individual. The women were convinced that God had to be in control for a successful birth. One woman said; ….*things to do with childbirth, it is God’s grace* and others affirmed:

*You must believe in yourself and believe in God. ….for me during birth, I said yes I will do it… I became firm and pushed the baby. Believing in yourself helps you to push the baby* (Zaharah, 24 years, mother of one)*.*

“*It’s not an easy thing but when you are determined, God can be by your side, yah then you will get a child”* (Irene, a 26-year-old first time mother).

As some women believed childbirth had a strong bearing on one’s personal effort put in during labour, to others birthing without complications was perceived and directly associated with “being strong and brave” as accounts below indicated:

*.... people used to consider me as a weak person and thought I would not manage to give birth by myself… but I demonstrated that I am strong*, *(She stressed the point very happily, entire group went into laughter)…I pushed the baby without any problem* (Amina, an 18 year old, primepara)

Anna, a 28-year-old first time mother also added:

“….. *others when they start to push, they push and even die. I had to push and I saw the baby; then what I concluded is that i am brave because it is not easy there”*

#### Societal and cultural interpretation of childbirth experiences

When women were asked to comment on how society perceived the different birth experiences they had gone through, the responses were cross cutting despite mothers being from different tribal and cultural backgrounds. The socio- cultural interpretations were mainly bent on the type of birth a woman went through. A mother was considered a strong woman if she gave birth well (vaginally) and the operated were regarded bewitched, failures or weak women.

Caroline, who had 3 consecutive operations said:

*The Gisu’s themselves think that when you go for an operation, …. you are bewitched. …you are unable to have children, unable to be a real housewife; … not a good house woman to make a child for somebody.*

Irene, a first- time mother who delivered by caesarean birth was insulted by the community members as she explained:

*Culturally, to the illiterate, they assume whoever goes for an operation failed to push. You are a weak woman. So, they were insulting me “eeeh she had just fattened, but you see, she failed to push the baby”.*

## Discussion

The study explored and presents the childbirth experiences among postnatal mothers and the meaning attributed to such experiences. The themes that emerged majorly focused on childbirth pains and management, institutional care and support, social support, childbirth fears and the meaning attributed to their childbirth experiences.

### Childbirth pains and management

The intensity of labour pains, the duration and patterns gave women different birth experiences and responses. The varied experiences were a reflection that each labour and birth a woman goes through is a unique experience even to women who have given birth before [27].This calls for proper assessment and understanding of the individual intrapartum needs of a woman by the health care providers and emotional support by the birth companion for a positive childbirth experience [28]. It was noted that people in the communities orient women to consider labour pains natural and normal regardless of their nature and this is intended to build courage and self-confidence of women during birth. Similarly, in a study done in Ghana, a first time mother was told in advance by her mother that labour was painful but should harden and remain strong [29]. Although women experienced prolonged and exhaustive labour pains with moments of confusion, inability to move, backs being felt in pieces and feelings of despair, divergent feelings of yes and no need regarding biomedical pain relief administration during childbirth were elicited. The women that were not in favour of the administration of drugs for pain relief simply felt birth pains were natural and medicine could not stop them. These expressions were of no surprise given that use of drugs to control pain during childbirth is still a rare occurrence in the country’s health care system like it is reported in other settings [29]. The evidence that none of the mothers received medication for pain relief despite a few of them expressing desire to have it, was a clear indication of limited access to such interventions. The women posited that without pain a baby could not come out, an indication of a knowledge gap on use of drugs for pain relief during labour. This calls for orientation of women on pain management options to empower them demand for their right of a pain free labourexperience as also recommended by the WHO intrapartum care model of 2018 [28].

### Institutional care and support

The women’s experiences of care and support varied from positive to negative and non-satisfying. The majority of women had given birth from the Regional Referral Hospital apart from one woman who had given birth from a private maternity centre. The needs and expectations of mothers in labour may seem to be many, however from this study it became apparent that giving attention and listening to women; good interpersonal communication and involving them in their care plans were key; the provision of physical and psychological support and competence in the management of complications were all paramount. The extent to which care met the individual expectations of mothers influenced the individual comments which were either reflective of a positive or negative birth experience. The women who received attention, good care and support expressed comfort and contentment of the hospital services and built high degree of confidence in the service providers and themselves. In a study done in Australia, women who were under supportive midwives felt contented with their childbirth experiences as opposed to those who were not and felt frustrated [27]. Similar findings have been reported in studies done in Iceland and India, where kindness and supportiveness to women in labour were strongly emphasized and closely related to a satisfying childbirth experience [30, 31]. On the contrary, in this study, women who received little attention, minimal technical, physical and psychological support had their birth experiences non-notable. And as such women lost self-confidence, distanced themselves from the care providers and some built bitterness against them. It was also noted that lack of provision of adequate information about labour processes and progression, poor communication and low involvement of mothers in their plans of care deprived them of full situational control and increased anxiety, stress and other emotional tendencies. Such findings are strongly backed by studies [27, 32], in which involvement of mothers in decision making during childbirth increased self-confidence, self-esteem, feelings of being in control and felt they had accomplished a task. These aspects of care are far reaching [28] that a mother, who misses them, will register a gap and a negative childbirth experience. This therefore calls for a positive attitude of a service provider to enhance and promote these indispensable needs during childbirth.

### Social support

Support from family members (mums, sisters, mothers in-law, aunties, husbands) and friends was strongly noted during this study. Escorting women to the birth places, provision of food and other requirements; rubbing the back, helping the mother to move to and forth were among the physical support given. Psychologically, they were supported through consolation and counseling and orientation on labour pains before or during labour. Social support in this study was strongly noted as a source of comfort, encouragement and hope to the mothers as strongly supported in other studies [33].

The presence of the male partner in the labour ward and his proximity to the spouse was appreciated by some women while others felt comfortable when their spouses were at a distance for fear of shifting blame to their male partners as trouble causers. Such divergent views and feelings about the men’s availability in labour wards have been cited in [34] in which some women expressed fear of their partners being impolite and non-loving to them while others were comfortable and in agreement with the practice. In Uganda, the practice of a female partner being with her spouse in a labour suite is just starting (mainly in private hospitals) and thus it is new to most women and men contrary to the developed world where the culture is deeply embedded in their care [35]. In a Swedish study [36] men from other countries appreciated the Swedish culture that transformed them from being “men at a distance” and women at “close proxy” to “a family” at the centre of every family member’s health concerns including childbirth. In this study, most participants had given birth from a government hospital and like most other government health facilities in this country, it had a general labour ward whose admission capacity was up to six mothers at a single moment. This minimized the space for a single laboring woman whose privacy was fragile and only protected by a curtain in between the beds. This further explains the low number of male partners and their low levels of involvement in labour processes for their female partners especially in the labour wards. Similar findings of a non-supportive environment for male involvement, have been reported in a study done in Mulago hospital [37].The findings of the study clearly indicated that mothers in labour currently have two schools of thought regarding the presence of male partners in labour suites. This calls for more research in this area to fully understand the factors that influence male partner involvement in labour and birth processes.

### Childbirth fears

The fears of childbirth reported in this study were majorly associated with the uncertainties of birth outcomes especially women who underwent an operation for they were not sure of the lives of the baby and self; or whether it would result in complications. Similarly, Corbett and Callister [30] found out that the Tamil Nadu women had fear of “the unknown” as they could not imagine the birth pains, and also feared caesarean birth leading to anxiety. In a Taiwan study [38] the caesarean births augmented more the risk of stress compared to women who gave birth vaginally and it was thought to be associated with hormonal differences or lack of self-confidence. In a Turkish study, the complications greatly contributed to birth fears [39]. These fears are further justified by Harrison and Goldenberg [40] who found out that complications like heamorrhage, embolism, hypovolemic shock, pre-eclampsia and eclampsia contributed to poor or severe maternal outcomes especially in resource limited geographies among caesarean births.

The non-satisfying childbirth experience due to the negative attitudes and practices of the health care providers frightened women and as such they vowed never again to give birth in the public hospitals. Furthermore, the stories the mothers heard from other women about the care and the practices of health care providers in hospital, caused some women to resist moving to hospital with the eventual decision to give birth with the TBA. This puts a woman further at risk and undermines the quality of the outcomes of labour given the limited level of knowledge and skills of the traditional birth attendant [41].Although labour pains were significantly noted by almost all women, to a lesser extent they were reported to be a source of fear possibly due to the social orientation of women about labour. These findings are contrary to what was found out in a Turkish study where labour pains was the most feared component of childbirth [42].Generally,fear caused stress and anxieties to mothers, both of which are factors that have been found to distort contractions and prolong labour and render one’s birth experience negative [33]. Overall, it is important to note, that creating a conducive birthing environment, free of fear, is key in promoting a sense of trust and confidence both in the health care providers and in theservices they offer for a positive childbirth experience.

### Meaning attached to childbirth experiences

The meaning of the childbirth experiences captured was two folded. The first one was – the mother’s personal interpretation of her own birth experience and two was how other people in the community interpreted the birth experience she went through. The women drew personal meanings and lessons of their birth experiences mainly from the nature of labour pains they had, the treatment by the service providers, the complications experienced and the labour outcomes. According to their accounts, women felt it was important and safer to deliver in a hospital especially those who had given birth at home and those who got complications like severe bleeding.Such lessons learnt could be helpful to a woman to subsequently make rational decisions regarding the birthing place. On the contrary, in a study done among Hmong women of Vietnam, majority of participants preferred a home birth for fear of loss of control over birth care decisions, limited comfort and loss of the placenta for cultural rituals [30].Findings also revealed that self-determination, self-confidence, self-belief and perseverance contributed to their successful childbirth. Although these ideologies may sound non- objective, they are important to note for they provide a basis for further assessment of their underlying causes from which objective interventions can be designed to improve childbirth. Self-belief and self-confidence have been found to promote and were closely related to positive childbirth experiences [43]. The women associated a normal vaginal birth (a birth free of complications) to braveness and being strong as they withstood and tolerated the birth hardships especially contractions. Such beliefs are important to understand by a health care provider so as to provide appropriate counseling to those who may experience complications during childbirth and those who undergo cesarean birth to prevent frustration, loss of self-esteem and feelings of inadequacy.

The cultural and social interpretations of childbirth experiences were the perceived meanings of childbirth by other people. Given the fact that these mothers were living in the same communities, it was speculated that the participants knew or had heard what people say about their birth experiences. According to the accounts of mothers, the majority of meanings were drawn from the type of birth one underwent. Culturally, every woman is expected to be strong, endure with pain and give birth vaginally. So, if a woman gave birth by caesarean, she would be considered bewitched, failed to push (a failure), weak and a woman not good to make a housewife. These findings might be so because in many African cultures, what goes wrong is thought to be an influence of evil spirits. They also believe in big number of children yet a mother, who is operated right from the first child, can safely produce four. Such interpretations may cause loss of self-esteem leading to poor adjustment to motherhood [43]. Understanding the cultural interpretations of childbirth experiences helps a health care provider to provide emotional support to the sufferers and their families and generally for the decision makers to design interventions that can address knowledge gaps regarding birthing options.

### Limitations

Childbirth experiences were got from mothers of parity five and below despite trying hard to look for those ones with parity above five. These possibly would have given different stories regarding childbirth. Literature from Africa on childbirth experiences and their meaning to mothers was limited giving a narrow point of reference when discussing the results in regard to the African context. Therefore, we recommend that subsequent similar or related studies look at settings like Latin America for a broader and closer contextual comparison. The socio cultural interpretations of the childbirth experiences may not fully reflect the views of all the other people in the studied community given the narrow source of the views.

## Conclusion

Childbirth experiences were unique and elicited unique feelings, responses and challenges to individual mothers, thus the need for the proper assessment and understanding of women for personalized care. It is important to note that pain management during labour is a necessity and a right of a woman to experience a pain free labour and birth for a positive childbirth outcome for those who embrace it. Creating a good birthing environment with the health care providers that are competent, compassionate and supportive to their clients, builds a sense of trust, and confidence in them and their services and the institution at large which promotes the general wellbeing of a woman at birth. Understanding the societal perceptions of childbirth experiences provides a basis for identifying and designing interventions to support the victimized mothers and the communities to understand childbirth.

## Operational definitions

- **Childbirth experiences:** Is an individual woman’s life event that incorporates interrelated subjective psychological and physiological processes, influenced by social, environmental, organizational and policy contexts [1].
- **Childbirth**: In this study, childbirth referred to labour and birth.
- **Experiences:** the personal lived through encounter of the phenomenon.
- **Mothers:** women who had been pregnant and delivered.
- **Meaning:** were the individual and subjective interpretations of a situation or a happening (her childbirth experiences) by the mother.
- **Postpartum period:** Is the period from one hour following the delivery of the placenta up to six weeks (WHO, 1998). However, in this study the postpartum period referred to the period from one hour after the birth of the placenta up to eight weeks or two months.

## Abbreviations

DHO: District Health Officer
FGD: Focus Group Discussion
M. o. H: Ministry of Health
SSI: Semi Structured Interviews
WHO: World Health Organization
YCC: Young Child Clinic
